# The Change of Cytokines and Gut Microbiome in Preterm Infants for Bronchopulmonary Dysplasia

**DOI:** 10.3389/fmicb.2022.804887

**Published:** 2022-03-21

**Authors:** Zhenjie Zhang, Jingjing Jiang, Zhenghong Li, Weilin Wan

**Affiliations:** ^1^Department of Pediatrics, Peking Union Medical College Hospital, Chinese Academy of Medical Sciences and Peking Union Medical College, Beijing, China; ^2^Department of Pediatrics, State Key Laboratory of Complex Severe and Rare Diseases, Peking Union Medical College Hospital, Chinese Academy of Medical Sciences and Peking Union Medical College, Beijing, China

**Keywords:** bronchopulmonary dysplasia, cytokines, gut microbiome, preterm infants, inflammation

## Abstract

**Background:**

Bronchopulmonary dysplasia (BPD) is a devastating form of chronic lung disease that develops in preterm infants. BPD is speculated to arise from abnormal inflammatory responses, which is related to the composition of commensal microbiota, leading us to hypothesize that BPD susceptibility could be influenced by gut microbiota through inflammatory responses. This study is aimed to detect cytokines and the differences in fecal gut microbial composition in the BPD patients.

**Methods:**

Between June 2018 and June 2020, preterm infants born at gestational age ≤30 weeks were recruited. The clinical data of infant characteristics were collected. On days 3–7 and 14–28 after birth, fresh stool samples and serum were collected. The gut microbiota composition between the BPD group and controls was detected by 16S rRNA sequencing. On days 3–7 and days 14–28, ten cytokines including IL-1β, IL-2, IL-4, IL-6, IL-8, IL-10, IL-12p70, IL-13, IFN-γ, and TNF-α were detected in the serum.

**Results:**

This study enrolled 38 preterm infants; the number of preterm infants in the BPD group and control group was, respectively, 18 and 20. The gestational age (27.4 ± 1.5 weeks vs. 29.5 ± 0.9 weeks, *p* = 0.000) and birth weight (971 ± 240 g vs. 1262 ± 335 g, *p* = 0.000) of the BPD group were lower than those of the control group. The present study found that the BPD group had high levels of IL-1β, IL-4, IL-6, IL-8, and TNF-α, whereas IL-10 was decreased. The Shannon diversity index of the BPD group was lower. The relative abundances of Proteobacteria in BPD group increased significantly from days 3–7 to days 14–28, while the Firmicutes was decreased. On days 14–28, the relative abundances of Proteobacteria in BPD group were significantly higher than those in the control group, while the Firmicutes was lower.

**Conclusion:**

Bronchopulmonary dysplasia could be influenced by gut microbiota through inflammatory responses. More studies are needed to explore the imbalance of cytokines and microbiome in BPD infants and whether it could be reversed by probiotics. This study provided a novel perspective for treating BPD.

## Introduction

Infants born preterm are at high risk for developing bronchopulmonary dysplasia (BPD), a chronic lung disease of prematurity. The adverse effects of early exposure of the lung to the extrauterine environment result in BPD, as well as the need for mechanical ventilation and oxygen delivery (Bancalari et al., 2003). Normal lung development can be disrupted by these stimuli. Thus, further research of risk factors and mechanisms that promote inflammation and impair lung development after preterm birth is critical to instituting better management strategies.

The pathogenesis of BPD is recognized to be multifactorial, and both pre- and post-natal conditions have potential impacts on its development. Prior studies indicate that the gut microbiota significantly influences the immune development of infants. Our previous research found that the cytokines of IL-4, IL-6, IL-8, and TNF-α increased and were considered as high-risk factors for BPD (Zhang et al., 2021). Our next research plan is to study whether BPD is related to gut microbiota.

Previous evidence suggests that the gut microbiota plays a key role in the immune development of newborns (Milani et al., 2017). Ecological shifts in the gut microbiome, domination by potentially pathogenic bacteria, and reduction in microbial diversity are associated with augmented inflammatory responses and immune system disorders, which, in turn, modulate lung development (Budden et al., 2017). The gut microbiota in very low birth-weight (VLBW; born weighing < 1,500 g) preterm infants is different from that of healthy term-born infants (Warner et al., 2016), and prolonged antibiotic use in VLBW preterm infants can increase the risk of developing BPD (Cantey et al., 2017). The current study indicates that immune homeostasis can be maintained by normal intestinal and lung commensal microbiota, and the disruption of them may lead to abnormal inflammation and participate in the pathogenesis of BPD (Pammi et al., 2019), leading us to hypothesize that BPD could be influenced by gut microbiota through inflammatory responses.

Therefore, this study focused on premature infants to detect the differences in gut microbiota and cytokines in the early life of BPD patients.

## Materials and Methods

We conducted a prospective study, which was approved by the Ethics Institutional Review Board of Peking Union Medical College Hospital (PUMCH). The parents of each infant signed informed consent who were agreeable to the use of feces and residual serum from routine testing for detection.

### Study Population

Inclusion criteria: (1) preterm infants born at gestational age ≤30 weeks from June 2018 to June 2020; (2) preterm infants admitted to the NICU at PUMCH within 3 days after birth; (3) the parents agreed to participate in the study and signed the informed consent.

Exclusion criteria: (1) infants had severe infection, shock, inherited metabolic diseases, or congenital malformations; (2) infants who died or were transported to other hospitals.

The diagnosis of BPD was as follows: 36 weeks post-menstrual age or discharge to home, whichever came first, while treatment with oxygen >21% for at least 28 days plus, as defined by the National Institutes of Child Health, the Human Development/National Heart, Lung, and Blood Institute, and the Office of Rare Diseases (Jobe and Bancalari, 2001). The infants with BPD were assigned to the BPD group, while the control group comprised infants without BPD.

### Data Collection

The basic maternal characteristics were collected, including premature rupture of membrane (PROM), antenatal steroids, and delivery mode, among others. The basic infant characteristics were collected, including gestational age, birth weight, neonatal respiratory distress syndrome (NRDS), Apgar score, duration of mechanical ventilation (in days) (MV), and patent ductus arteriosus (PDA). Clinical data were prospectively collected by one researcher and verified by another.

### Inflammation Biomarkers

The parents or legal guardians of eligible infants were contacted as soon as possible after birth (usually < 72 h). To limit invasiveness, this study only analyzed the residual serum that was routinely tested weekly in the hospital laboratory. Serum cytokines were measured 3–7 days and 14–28 days after birth. Our study detected ten cytokines, including IL-1β, IL-2, IL-4, IL-6, IL-8, IL-10, IL-12p70, IL-13, IFN-γ, and TNF-α.

Our laboratory had adopted strict experimental procedures to minimize protein degradation. The blood collected in BD Microtainers (Becton Dickinson, Mississauga, ON, Canada) was spun within 1 h to remove cell fraction, and the remaining serum was immediately stored at -80°C. Serum was collected, MSD technology was used, and U-PLEX Biomarker Group 1 (Human) Multiplex Assay (K15049D) was used to detect cytokine protein levels. MESO QuickPlex sq120 machine and MSD Discovery Workbench version 4.0.12 were used, respectively, for detection and data analysis.

### Sample Preparation and Sequencing

Each subject collected fresh stool samples 3–7 days and 14–28 days after birth. No antibiotics were used before sample collection for 3 days.

#### Extraction of Genome DNA

The CTAB method was used to extract the total genomic DNA of the sample. Agarose gel (1%) was used to monitor DNA concentration and purity. According to the DNA concentration, 1 ng/μl was diluted with sterile water.

#### Amplicon Generation

16S rRNA genes of distinct regions 16S V4 were amplified used specific primers 515F (5′ GTGCCAGCMGCCGCGGTAA 3′) and 806R (5′ GGACTACHVGGGTWTCTAAT 3′) with the barcode.

All PCR reactions were carried out in a 30-μl reaction, 15 μl Phusion High-Fidelity PCR Master Mix (New England Biolabs, Ipswich, MA, United States), 0.2 μM forward and reverse primers, and about 10 ng template DNA. Thermal cycle: initial denaturation at 98°C for 1 min, denaturation at 98°C for 10 s, annealing at 50°C for 30 s, and extension at 72°C for 30 s, totaling 30 cycles. Finally, the temperature was kept at 72°C for 5 min.

#### PCR Product Mixing and Purification

An equal volume of 1× loading buffer (containing SYB green) was mixed with the PCR product, and electrophoresis detection was performed on a 2% agarose gel. The PCR products were mixed in an equal density ratio. The mixed PCR products were purified with GeneJET Gel Extraction Kit (Thermo Scientific, Vilnius, Lithuania).

#### Library Preparation and Sequencing

According to the manufacturer’s recommendations, the Ion Plus Fragment Library Kit 48 rxns (Thermo Scientific, Vilnius, Lithuania) was used to generate sequencing libraries. Qubit@2.0 Fluorometer (Thermo Scientific, Vilnius, Lithuania) was used to evaluate the quality of the library. Finally, the library was sequenced on the Ion S5 XL platform, and 400 bp/600 bp single-ended reads were generated.

#### Data Analysis

##### Single-End Reads Quality Control

(1) Data split. Single-ended reading was based on the unique barcode of the sample and is truncated by cutting the barcode and primer sequence. (2) Data filtering. According to the Cutadapt (V1.9.1^1^) quality control process, the original reads were quality filtered under specific filtering conditions to obtain high-quality clean reads. (3) Chimera removal. The reads were compared with the reference database (Silva database^2^) using UCHIME algorithm (UCHIME Algorithm^3^) to detect chimera sequences, and then the chimera sequences were removed. Then the Clean Reads finally obtained.

##### Operational Taxonomic Unit Cluster and Species Annotation

(1) Operational taxonomic unit (out) production. Sequences analysis were performed by Uparse software (Uparse v7.0.1001^4^). Sequences with ≥97% similarity were assigned to the same OTUs. Representative sequence for each OTU was screened for further annotation. (2) Species annotation. For each representative sequence, the Silva Database (see text footnote 2) was used based on Mothur algorithm to annotate taxonomic information. (3) Phylogenetic relationship construction. To study the phylogenetic relationship between different OTUs and the differences in dominant species in different samples (groups), MUSCLE software (version 3.8.31^5^) was used for multiple sequence alignment. (4) Data normalization. The abundance information of OTUs was normalized using the serial number standard corresponding to the sample with the least sequence. Subsequent alpha diversity analysis was based on this output normalized data.

##### Alpha Diversity

Alpha diversity is applied in analyzing complexity of species diversity for a sample through Shannon index. It was calculated with QIIME (version 1.7.0) and displayed with R software (version 2.15.3).

### Statistics

SPSS version 25.0 was used for statistical analysis. Normally distributed quantitative data were expressed with mean ± standard error ($\bar{X}$ ± S). The two groups of data were compared by *t*-test. Quantitative data that did not conform to the normal distribution were described by the median and interquartile range [M (P25–P75)], and comparison between groups was tested using the rank sum test. A two-way ANOVA was used for comparing the composition of the overall microbiome between different groups. Non-parametric Mann–Whitney *U*-tests were used to analyze continuous data. The qualitative data were described by frequency and rate. Pearson’s χ^2^ test or Fisher’s exact test was conducted. Logistic regression was used to analyze the independent risk factors of BPD. A *p*-value < 0.05 was considered statistically significant.

## Results

### Baseline Data

From June 2018 to June 2020, forty-two cases of premature infants with gestational age ≤30 weeks were born in our hospital. Four cases of premature infants were excluded due to insufficient detection of residual serum cytokines. A total of 38 premature infants were included in this study; the number of preterm infants in the BPD group and control group was, respectively, 18 and 20. There were no premature infants with small gestational age. The start time of enteral feeding was within 12 h after birth. Because there was a breastmilk bank in our hospital, all premature infants were breastmilk feeding.

The gestational age of the BPD group (27.4 ± 1.5 weeks) was lower than that in the control group (29.5 ± 0.9 weeks) (*p* = 0.000). The birth weight of the BPD group (971 ± 240 g) was lower than that in the control group (1,262 ± 335 g) (*p* = 0.000). It could be seen from Tables 1, 2 that the incidence of PDA in the BPD group was higher; the mechanical ventilation time and hospital stay of the BPD group were significantly higher than those of the control group.

**TABLE 1:** The basic infant characteristics between the BPD and control groups.

	BPD group (*n* = 18)	Control group (*n* = 20)	*P*-value
Male gender *n* (%)	11 (61%)	10 (50%)	0.492
Birth weight (grams) $\bar{\text{X}}\pm \text{S}$	971 ± 240	1262 ± 335	0.000
Gestational age (weeks) $\bar{\text{X}}\pm \text{S}$	27.4 ± 1.5	29.5 ± 0.9	0.000
Apgar 1 min $\bar{\text{X}}\pm \text{S}$	7.8 ± 2.1	8.4 ± 1.6	0.296
Apgar 5 min $\bar{\text{X}}\pm \text{S}$	8.8 ± 1.3	9.5 ± 0.8	0.060
Apgar 10 min $\bar{\text{X}}\pm \text{S}$	9.1 ± 0.8	9.7 ± 0.6	0.469
NRDS *n* (%)	18 (100%)	17 (85%)	0.087
Patent ductus arteriosus (PDA) *n* (%)	15 (83.3%)	9 (45%)	0.014
Late-onset neonatal sepsis *n* (%)	7 (38%)	8 (40%)	0.735
Duration of mechanical ventilation (days) $\bar{\text{X}}\pm \text{S}$	32.5 ± 17.0	12.9 ± 7.6	0.000
Duration of invasive ventilation (days) $\bar{\text{X}}\pm \text{S}$	6.1 ± 5.0	0.4 ± 0.9	0.000
Intravenous hormone *n* (%)	9 (50%)	1 (5%)	0.002
Length of hospital stay (days) $\bar{\text{X}}\pm \text{S}$	61.2 ± 16.9	42.2 ± 10.5	0.000
Corrected gestational age at discharge $\bar{\text{X}}\pm \text{S}$	36.3 ± 1.5	35.1 ± 1.0	0.000
Duration of parenteral nutrition: $\bar{\text{X}}\pm \text{S}$ (days)	42.1 ± 12.1	25.6 ± 10.2	0.000

*N represents the number of patients.*

**TABLE 2:** The basic maternal characteristics between BPD and control groups.

	BPD group (*n* = 18)	Control group (*n* = 20)	*P*-value
Maternal age (years) $\bar{\text{X}}\pm \text{S}$	33.7 ± 6.8	34.0 ± 4.9	0.056
Cesarean section *n* (%)	14 (77.8%)	17 (85%)	0.142
Antenatal steroids *n* (%)	12 (66.7%)	13 (65%)	0.132
Premature rupture of membranes (PROM) *n* (%)	6 (33%)	11 (55%)	0.180
Maternal antibiotic treatment *n* (%)	6 (33%)	5 (25%)	0.572

*N represents the number of patients.*

Logistic regression analysis was conducted in this study. Gestational age, birth weight, and PDA were used as independent variables. The data indicated that gestational age < 28 weeks (OR = 5.528, *p* = 0.000) and birth weight < 1,000 g (OR = 3.416, *p* = 0.037) were high-risk factors for BPD.

### Inflammatory Cytokine

A total of 760 times of cytokines were evaluated from 76 blood samples. The 10 cytokines that were detected include IL-1β, IL-2, IL-4, IL-6, IL-8, IL-10, IL-12p70, IL-13, IFN-γ, and TNF-α (see Table 3). On days 1–7, there were no significant differences between the BPD and control groups. On days 14–28, IL-1β, IL-4, IL-6, IL-8, and TNF-α were significantly increased in the BPD group, whereas IL-10 was decreased (Table 3).

**TABLE 3:** The cytokines of the patients.

	BPD group (*n* = 18)	Non-BPD group (*n* = 20)	*P*-value
**CYTOKINES (pg/ml) M (P25–P75) Days 3–7**			
IFN-γ	9.0(6.8−18.0)	8.8(6.8−15.2)	0.860
IL-10	2.5(2.1−3.1)	3.0(2.1−7.3)	0.081
IL-12p70	1.2(1.0−1.4)	1.05(0.9−1.2)	0.232
IL-13	6.1(4.6−8.9)	7.8(6.2−9.7)	0.095
IL-1β	1.5(1.1−1.9)	1.9(1.1−2.0)	0.233
IL-2	2.1(1.7−2.4)	1.9(1.5−2.1)	0.206
IL-4	0.4(0.2−0.5)	0.3(0.1−0.5)	0.300
IL-6	6.2(5.8−6.9)	5.3(5.1−6.8)	0.193
IL-8	175(157−181)	157(146−166)	0.067
TNF-α	0.6(0.4−0.9)	0.5(0.2−0.6)	0.128
**CYTOKINES (pg/ml) M (P25–P75) Days 14–28**			
IFN-γ	9.5(6.9−18)	12.5(7.2−16.2)	0.907
IL-10	2.3(2.1−2.9)	2.9(2.1−7.3)	0.038
IL-12p70	0.7(0.4−1.1)	0.9(0.7−1.1)	0.240
IL-13	8.1(7.9−9.3)	9.0(7.9−10.8)	0.437
IL-1β	2.9(2.1−3.4)	2.0(1.5−2.4)	0.001
IL-2	2.0(1.8−2.6)	1.8(1.3−2.1)	0.089
IL-4	0.9(0.3−1.2)	0.3(0.2−0.5)	0.001
IL-6	8.7(7.9−9.8)	5.5(4.6−6.0)	0.000
IL-8	482(436−547)	267(235−295)	0.000
TNF-α	1.1(0.8−1.5)	0.6(0.4−1.0)	0.001

### The Microbiome Diversity of Shannon Index Between Infants With and Without Bronchopulmonary Dysplasia

The microbiome diversity of Shannon index on days 3–7 was lower in the BPD groups [M (P25–P75), 3.41 (3.14–3.87)] compared with the control groups [4.38 (4.25–4.68), *p* = 0.00]. The Shannon index on days 14–28 was lower in the BPD groups [3.71 (3.34–3.83)] compared with the control groups [4.58 (4.35–5.11), *p* = 0.00].

### Evolution of the Microbiome Differs Between Infants With and Without Bronchopulmonary Dysplasia

**FIGURE 1:**
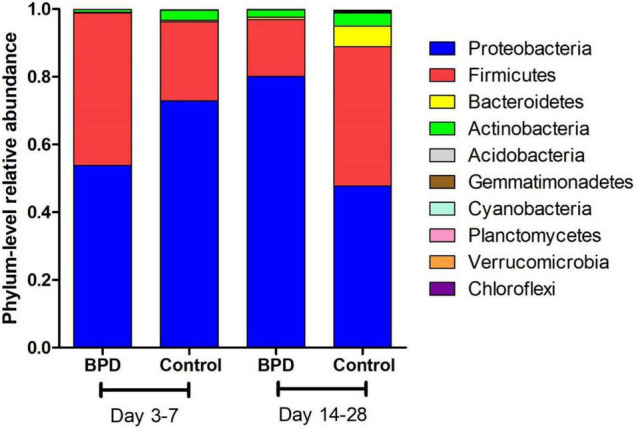
The mean relative abundances of major microbiome (Top 10) of both groups at the phylum level on days 3–7 and 14–28.

Figure 1 presents the mean relative abundances of major microbiome (Top 10) of both groups at the phylum level on days 3–7 and 14–28. Four phyla (Proteobacteria, Firmicutes, Bacteroidetes, and Actinobacteria) dominated the fecal microbiota in both groups on days 3–7 and 14–28.

A two-way ANOVA was used for comparing the composition of the overall microbiome between different groups. It found that these two couples had statistical difference: days 3–7 BPD group vs. days 14–28 BPD group (*p* = 0.03); days 14–28 BPD group vs. days 14–28 control group (*p* < 0.001). The phylum-level differences in microbiome between both groups were analyzed by *t*-test. The relative abundances of Proteobacteria of BPD group increased significantly from days 3–7 to days 14–28 (53.9 vs. 80.1%, *p* = 0.032). On the contrary, the relative abundances of Proteobacteria of control group decreased from days 3–7 to days 14–28, but not significantly (72.9 vs. 47.7%, *p* > 0.05). The relative abundances of Firmicutes of BPD group decreased significantly from days 3–7 to days 14–28 (45 vs. 16.7%, *p* = 0.021). On the contrary, the relative abundances of Firmicutes of control group increased from days 3–7 to days 14–28, but not significantly (23.4 vs. 41.2%, *p* > 0.05).

On days 3–7, no significant difference in microbiome was found between BPD group and control group. On days 14–28, the relative abundances of Proteobacteria of BPD group were significantly higher than those in the control group (80.1 vs. 47.7%, *p* = 0.001), and the relative abundances of Firmicutes of BPD group were significantly lower than those in the control group (16.7 vs. 41.2%, *p* = 0.016).

**FIGURE 2:**
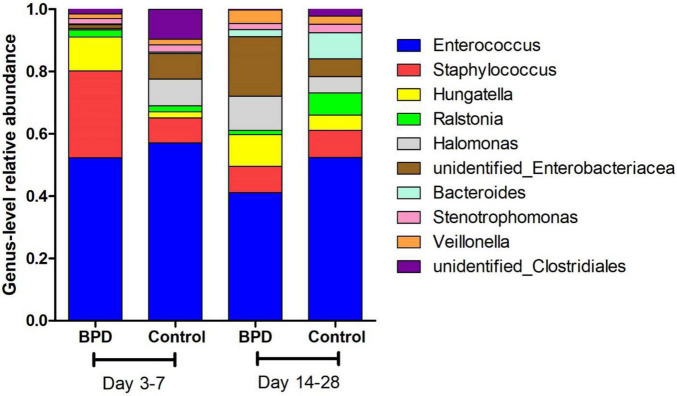
The mean relative abundances of major microbiome (Top 10) of both groups at the genus level.

Figure 2 presents the mean relative abundances of major microbiome (Top 10) of both groups at the genus level. The differences in microbiome at genus level between groups were analyzed by *t*-test. The relative abundances of *Enterococcus* on days 14–28 BPD group and control group were, respectively, 41.1 and 52.4%. The difference was statistically significant (*p* = 0.049). Our study found that the relative abundance in BPD group on days 14–28 of *Enterococcus* was lower.

**FIGURE 3:**
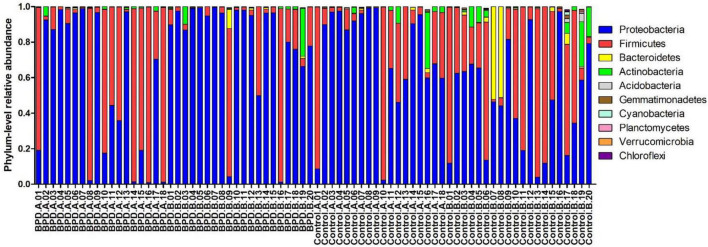
The mean relative abundances of major microbiome (Top 10) of every infant at the phylum level. BPD.A: The infants with BPD on days 3–7. BPD.B: The infants with BPD on days 14–28. Control A: The infants without BPD on days 3–7. Control B: The infants without BPD on days 14–28.

Figure 3 presents the mean relative abundances of major microbiome (Top 10) of every infant at the phylum level.

## Discussion

Over 50 years after its first description, BPD remains a devastating pulmonary complication in preterm infants (Northway et al., 1967). It was thought to involve ventilator- and oxygen-induced damage to an immature lung that results in an inflammatory response and ending in aberrant lung development with dysregulated angiogenesis and alveolarization (Lal and Ambalavanan, 2017). Significant morbidity and mortality were associated with this most common chronic lung disease. Past studies and our previous research had clearly shown that the inflammatory response and the elaboration of cytokines were associated with the development of BPD (Savani, 2018). A large body of evidence now suggested that the gut microbiota could influence immunity and inflammation systemically, including in the lung (Budden et al., 2017).

However, it was currently unclear whether the intestinal flora affects the susceptibility to BPD. To study whether changes in the intestinal flora are related to BPD, we used 16S rRNA gene sequencing to analyze the composition of the flora collected from 38 premature infants born at <30 weeks’ gestation. This article detected the change of cytokines and gut microbiome in preterm infants for BPD.

Gestational age and birth weight were one of the most important factors affecting the development of BPD (Capasso et al., 2019). In our study, the gestational age and birth weight of the BPD group were lower than those of the control group, while the incidence rate of PDA was higher in the BPD group. PDA could extend the time of mechanical ventilation after birth. Severe left-to-right shunt could cause lung congestion and edema, thus aggravating pulmonary vascular damage and lung inflammation (Clyman et al., 2021).

Bronchopulmonary dysplasia was related to the imbalance of pro-inflammatory factors in the body. A large number of pro-inflammatory factors were released, aggravating the damage to immature lungs and causing BPD. Our study found that the cytokines IL-1β, IL-4, IL-6, IL-8, and TNF-α were increased in the BPD group on days 14–28 significantly, whereas IL-10 was decreased. In the previous study, IL-1β, IL-6, TNF-α, and IL-10 cytokine levels were altered in the amniotic fluid (Yoon et al., 1997), cord blood (Rocha et al., 2012), and tracheal aspirate samples (Yilmaz et al., 2017) in BPD infants (Szpecht et al., 2017). Therefore, we speculated that these cytokines may be related to the lung injury of BPD infants. Cytokines changed dynamically at different time points. These cytokines were about the same in the early stage, but had obvious changes in the late stage.

Our study found that the duration of mechanical ventilation was significantly higher in the BPD group. Mechanical ventilation initiated an influx of neutrophils and macrophages into the alveoli (Bhandari, 2014). These cells produced cytokines, which could disrupt lung development and increase the risk of BPD (Koksal et al., 2012). Infants with BPD had persistently elevated pro-inflammatory cytokines (IL-1β, IL-6, and IL-8) in their tracheal inhalation and blood. In preterm lambs, 2 h of mechanical ventilation could initiate inflammation within the lungs resulting in similar upregulation of IL-1β, IL-6, and IL-8, the same biomarkers as infants that were ventilated (Kothe et al., 2019). Prolonged ventilation (3–4 weeks) in preterm lambs increased the infiltration of neutrophils and macrophages into the lungs, resulting in non-uniform inflation patterns and abnormal pulmonary vascular development, similar to that observed in infants who died from BPD (Papagianis et al., 2019).

**FIGURE 4:**
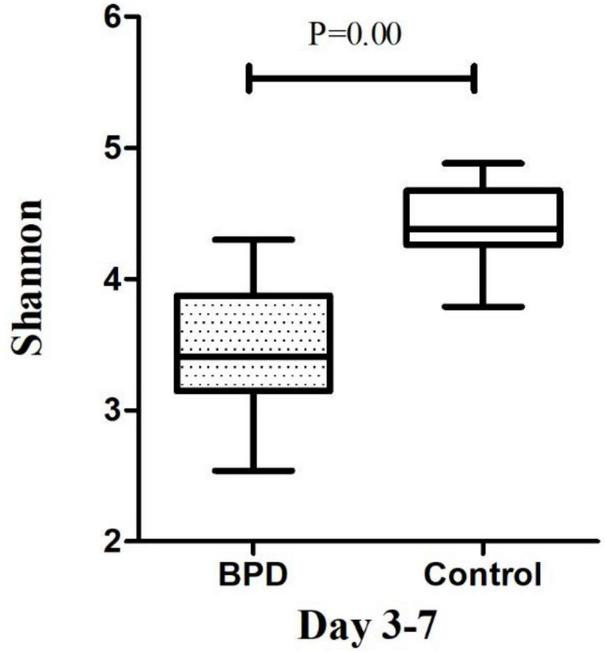
The microbiome diversity of Shannon index between BPD group and Control group on days 3–7. Shannon index [M (P25–P75): 3.41 (3.14–3.87) vs. 4.38 (4.25–4.68), *P* = 0.00].

**FIGURE 5:**
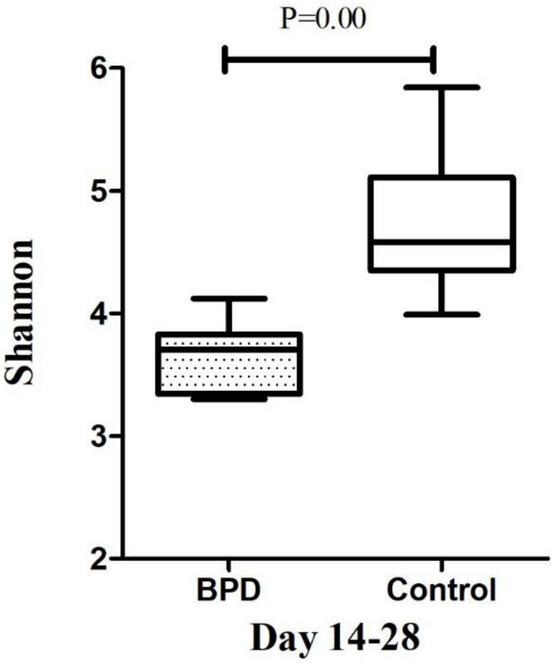
The microbiome diversity of Shannon index between BPD group and Control group on days 14–28. Shannon index [M (P25–P75): 3.71 (3.34–3.83) vs. 4.58 (4.35–5.11), *P* = 0.00].

Growing evidence supported the “gut–lung axis” theory, which suggested a potential connection between gut microbiota and lung homeostasis (Warner and Hamvas, 2015). Our study found that there was a significant difference in the Shannon diversity index of the gut microbial community between infants with and without BPD. The high value of Shannon index was usually the result of a species-rich and relatively evenly distributed community, while the low value of Shannon index usually reflected the low species abundance of the community, which might be dominated by a few dominant groups. Figures 4, 5 showed a difference in Shannon diversity index between the two groups. Shannon index showed that the microbial community diversity and the number of species in infants with BPD decreased significantly. This difference in diversity represented an apparent quantitative difference in infants, which may be a prediction of the development of BPD, and may actually be related to the mechanism.

Preterm infants, especially VLBW infants, were at a disadvantage in the development of a healthy microbiome. Facts have proved that the gut microbiota of premature babies reduces the diversity of microorganisms and increases the colonization of pathogenic microorganisms. In addition, the stability of the gut microbiome of preterm infants is lower than that of the term counterparts.

Previous studies had shown that Actinobacteria, Proteobacteria, Bacteroidetes, and Firmicutes were the four major phyla of the intestinal tract of premature infants (Gritz and Bhandari, 2015). According to our analysis of intestinal flora, these four phyla were also the dominant flora of our patients’ intestinal tract. We noted that the BPD group on days 14–28 had more Proteobacteria and less Firmicutes, and the differences were statistically significant. In contrast, infants who did not develop BPD continued to have a relatively stable and resilient microbiota, while the dominant species remained unchanged. In the control group, the diversity of microbes and the number of species were still high. As pointed out in other studies, the results of the study showed that the diversity of microbes is related to health status (Lohmann et al., 2014).

In the comparative study of the microbiota between the disease group and the healthy group, it was common to increase the abundance of Proteobacteria in the disease group (Rizzatti et al., 2017). It had also been suggested that the high abundance of Proteobacteria represents the microbial characteristics of dysbiosis and reflects the unstable structure of the intestinal microbial community (Shin et al., 2015). It seemed that the gut microbiome changed early in the course may be associated with BPD development.

The ecological changes of the microbiota were related to the increase of inflammation in other human systems. Specific patterns in the acquisition and composition of the microbiome and the altered epithelial barrier function in the intestinal tract of adults and premature infants were associated with inflammation (Dimmitt et al., 2010). However, because the immune system of premature infants was still in the mature stage, simple bacterial colonization might be sufficient to drive the inflammatory response, disrupt lung development, and lead to BPD. On the other hand, there might exist potential probiotics that were important for maintaining physiological homeostasis of lung. Our data exhibited potential for clinical application.

We hypothesize that the microbiome could influence BPD through cytokines; thus, it was speculated that the probiotics could alleviate BPD by recovering microbial composition. In an *in vitro* study, treatment with probiotics *Bifidobacterium* and *Lactobacillus* significantly inhibited the population of Proteobacteria, and increased the populations of Bacteroidetes and Actinobacteria. Furthermore, the probiotics inhibited expression of IL-4 in the mice but inhibited Th2 cell activation. They could also increase Treg cell differentiation such as IL-10 expression. Moreover, these also suppressed TNF-α expression in activated macrophages (Li et al., 2019). In another study, the combined probiotic (containing *L. plantarum* 20%, *B. longum* 40%, and *B. bifidum* 40%) treatment could strongly increase the abundance of Firmicutes, whereas that of Proteobacteria was significantly reduced in preterm infants, while it was accompanied by a decrease of IL-6 (Kim et al., 2019). Thus, we hypothesize that the microbiome could influence BPD by cytokines. Compared with the changes in cytokines and microbiome of BPD group in the present study, probiotics could induce completely opposite changes. We could suppose that the imbalance of cytokines and microbiome in BPD infants could be reversed by probiotics.

Our study had several limitations. Since the intestinal flora in this life stage was unstable, we paid attention to a modest number of infants (*n* = 38) and many repeated samples to assess whether the changes in the intestinal flora were related to BPD. The sample size limited our statistical power, and further studies with larger sample sizes now needed to confirm our findings. Due to our relatively limited sample size, we did not evaluate the relationship between microbiota and BPD subtypes. In addition, all infants were recruited from the neonatal intensive care unit of the same hospital, which might be a source of some common microorganisms. Reproducing our findings in a larger multicenter study was the key to the next step. Finally, we hypothesize that the microbiome could influence BPD by cytokines, and more studies are needed to verify this viewpoint.

## Conclusion

Bronchopulmonary dysplasia was a disease resulting from a combination of multiple factors. The gestational age and birth weight of the BPD group were lower than those of the control group. The present study found that the BPD group had high levels of IL-1β, IL-4, IL-6, IL-8, and TNF-α, whereas IL-10 was decreased. The Shannon diversity index of BPD group was lower. The relative abundances of Proteobacteria in BPD group increased significantly from days 3–7 to days 14–28, while Firmicutes was decreased. On days 14–28, the relative abundances of Proteobacteria in BPD group were significantly higher than those of the control group, while the Firmicutes was lower. More studies are needed to explore the imbalance of cytokines and microbiome in BPD infants and whether it could be reversed by probiotics. This study provided a novel perspective for treating BPD.

## Data Availability Statement

The original contributions presented in the study are included in the article/supplementary material, further inquiries can be directed to the corresponding author/s.

## Ethics Statement

The studies involving human participants were reviewed and approved by ethical approval: The experimental protocol was established, according to the ethical guidelines of the Helsinki Declaration and was approved by the Human Ethics Committee of Peking Union Medical College Hospital, Chinese Academy of Medical Sciences. The ethical review number was ZS-1552. Written informed consent to participate in this study was provided by the participants’ legal guardian/next of kin.

## Author Contributions

ZZ: formal analysis, methodology, and writing—original draft. JJ: data curation and investigation. WW: funding acquisition, methodology, and project administration. ZL: methodology, project administration, and writing—review and editing. All authors contributed to the article and approved the submitted version.

## Conflict of Interest

The authors declare that the research was conducted in the absence of any commercial or financial relationships that could be construed as a potential conflict of interest.

## Publisher’s Note

All claims expressed in this article are solely those of the authors and do not necessarily represent those of their affiliated organizations, or those of the publisher, the editors and the reviewers. Any product that may be evaluated in this article, or claim that may be made by its manufacturer, is not guaranteed or endorsed by the publisher.

## Footnotes

1
http://cutadapt.readthedocs.io/en/stable/

2
https://www.arb-silva.de/

3
http://www.drive5.com/usearch/manual/uchime_algo.html

4
http://drive5.com/uparse/

5
http://www.drive5.com/muscle/

## Abbreviations

BPD: bronchopulmonary dysplasia
MV: duration of mechanical ventilation
NICU: neonatal intensive care unit
NRDS: neonatal respiratory distress syndrome
PDA: patent ductus arteriosus
PROM: premature rupture of membrane
VLBW: very low birth weight.
